# Calcitonin gene-related peptide monoclonal antibodies and medication-overuse headache: stopping excessive pain medication is still necessary

**DOI:** 10.1055/s-0045-1809332

**Published:** 2025-07-28

**Authors:** Pedro Augusto Sampaio Rocha-Filho

**Affiliations:** 1 Universidade Federal de Pernambuco, Centro de Ciências Médicas, Área Acadêmica de Neuropsiquiatria, Recife PE, Brazil.; 2 Universidade de Pernambuco, Hospital Universitário Oswaldo Cruz, Serviço de Neurologia, Recife PE, Brazil.

**Keywords:** Headache, Headache Disorders, Secondary, Migraine Disorders, Tension-Type Headache, Therapeutics, Analgesics, Review

## Abstract

Medication-overuse headache affects 1 to 2% of the global population and is often associated with chronic migraine. This condition significantly impacts the lives of patients, as well as their families, and it poses a major economic burden due to lost productivity and medical costs. The present narrative review is part of a controversy session. We argue that reversing the behavior of overusing symptomatic pain medications is important for the treatment of this type of headache. To support this argument, the article reviews and critically analyzes the relevant literature on the subject.

## MEDICATION-OVERUSE HEADACHE

To diagnose an individual with a medication-overuse headache (MOH), they must have experienced a prior headache, typically primary, whose characteristics change and/or worsen significantly with the regular use of symptomatic pain medications, leading to a frequency of daily or almost daily headaches (≥ 15 attacks/month). 1 2 3 With a varied potential, 2 pain medications that can cause MOH include opioids, triptans, ergotamine, nonsteroidal anti-inflammatory drugs (NSAIDs), and common analgesics.

It is estimated that the prevalence of MOH in the general population is between 1% and 2%. 1 3 Despite being classified as secondary, the requirement of having a prior headache for its development raises the question of whether excessive use of medications is a risk factor for worsening of the primary condition. For instance, excessive use of symptomatic pain medications is known to worsen migraines. 4 Most patients with medication-overuse headaches have chronic migraines, and vice versa. 1 3

There is a consensus that prophylactic treatment for the primary headache should be initiated early as part of MOH treatment, emphasizing that addressing the primary condition is central to management of the secondary one. 5 6

Neuroimaging studies in this cohort reveal structural and functional alterations in the pain-processing areas of the central nervous system. These patients also exhibit dysfunction in their antinociceptive system. Furthermore, research on the pathophysiology of MOH indicates that excessive use of pain medication contributes to the development of central and peripheral sensitization, supporting the idea that it exacerbates primary headaches. 1 3

The group of headache patients with excessive use of pain medications is heterogeneous. Some individuals use medications because their headaches have worsened in frequency but do not have MOH. In these cases, even if this excessive use stops, there may be no improvement in headache symptoms. Another subset of patients displays substance abuse behaviors. Some may suffer from cephalalgiaphobia, where fear of pain leads them to use medication preemptively, even before headache onset. Additionally, the types of medications used vary. To account for this heterogeneity, some authors have suggested classifying MOH into “simple” and “complicated.” The “complicated” cases would include patients with concurrent mood, anxiety, or eating disorders; with a history of substance addiction; those who use multiple doses of symptomatic medications almost daily; and those with a history of MOH relapses. 7 8

Reversing the behavior of excessive medication use has always been considered essential for treatment. This reversal not only leads to significant improvements in headache frequency, intensity, and impact but also decreases anxiety and depressive symptoms. 6 9 10 Most research indicates a success rate of over 70% in reversing medication overuse; however, about ⅓ of patients may relapse. 6

An important aspect to discuss is the best strategy for reversing medication overuse. Carlsen et al. compared two detoxification strategies: stopping pain medications completely for 2 months ( *n* = 35) versus restricting their use to 2 days a week ( *n* = 37). Both groups showed improvement and had similar rates of MOH resolution. However, the reduction in headache days was significantly greater in the group that stopped all pain medications (primary outcome), and this group had a higher rate of reversal from chronic to episodic migraines at the 6- and 12-month follow-ups. 11

A clinical trial 12 is often cited as evidence that it is unnecessary to stop using symptomatic medications in patients with MOH. This study compared two strategies: prophylactic treatment combined with stopping symptomatic pain medications (allowing medications from other classes for up to 2 days/week) versus maintaining symptomatic medications alongside prophylactic treatment. There was no difference between the groups in the frequency of days with moderate to severe headaches (primary outcome). However, by the end of the 12-week follow-up, the group that stopped medication use experienced significantly less overuse and had fewer days of intake. 12 Furthermore, it has been demonstrated that reducing medication use differs from discontinuing medication entirely. 11

A recent open-label clinical trial 13 evaluated three approaches for treating MOH. The trial involved three groups: one underwent withdrawal of symptomatic pain medications combined with the initiation of prophylactic treatment ( *N* = 40), another received preventive treatment without withdrawing symptomatic pain medications ( *N* = 40), and the third underwent withdrawal of symptomatic pain medications alone ( *N* = 40). After the withdrawal of symptomatic drugs, the group that did not receive prophylactic treatment was later offered this treatment. Monoclonal antibodies (MAbs) targeting the calcitonin gene-related peptide (CGRP) pathway were not available when this study was performed. All three groups experienced a reduction in headache days compared with their baseline measurements, with no significant differences among them (primary outcome). However, at the end of the 6-month follow-up, the group that stopped their excessive medication use and received a prophylactic treatment demonstrated a significantly greater reversal from chronic migraine to episodic migraine, as well as a higher rate of recovery compared with the other two groups. 13

## Why the controversy?

The post hoc analyses of clinical trials evaluating MAbs (erenumab, galcanezumab, fremanezumab, and eptinizumab) for treatment of chronic migraine showed a greater reduction in the frequency of migraine days and in the use of symptomatic medications in the drug group than in the placebo group among those with medication overuse. 14 15 16 17 These findings led some members of the scientific community to argue that discontinuing symptomatic medications was unnecessary.

## Why we should continue advising on reversing the overuse of symptomatic pain medications

Addressing the modifiable risk factors of chronic migraine is an essential part of its treatment. 5 The use of MAbs has also proven to be effective for individuals with various chronic migraine risk factors. We acknowledge the importance of treating anxiety, depression, obesity, insomnia, and obstructive sleep apnea. Notably, overuse of symptomatic medications is a well-recognized risk factor for chronic migraine. Treatments such as topiramate, valproate, and botulinum toxin have demonstrated efficacy in managing migraine, even among those who overuse symptomatic medications. 18 19 20 21 However, there was no question whether we should change the behavior of overusing medications.

The post hoc analyses can be misleading, as the original studies were not designed to answer this research question. Thus, the heterogeneity of this group was not considered. In these studies, between 35 and 60% of patients who overused medications and received MAbs were able to reverse their overuse, compared with 18 to 46% in the placebo group. It is possible that some of those who improved did not have MOH and were simply overusing medications.

From another perspective, the number needed to treat (NNT) MAbs for chronic migraine is 4.5 to 11 patients. 22 Notably, 40 to 65% of those who overused medications and received MAbs did not reverse their behavior. 1 Combining medication overuse reversal with prophylactic treatment has been shown to be more effective than either approach alone. 13 The question that remains is whether more individuals would have improved if they had reversed their symptomatic medication overuse behavior.

Finally, we must address a more technical aspect regarding the study's design. Randomizing participants in clinical trials helps ensure that confounding factors are distributed evenly between groups, which is true for known and unknown variables. However, when subanalyses of studies are conducted, this benefit of randomization is lost, increasing the potential for bias due to the differential distribution of confounding factors between groups.

## What has changed?

Ceasing medication overuse has always been central to the treatment of MOH. For patients unable to discontinue use in an outpatient setting, hospital withdrawal was necessary. Now, there are effective medications even if overuse behavior isn't reversed. The International Headache Society, in its recent recommendations for migraine prophylactic treatment, advises individuals with MOH to reduce or stop the overuse of symptomatic medications. However, they note that for MAbs, topiramate, or botulinum toxin, immediate withdrawal or reduction may not be required. 23 This offers an opportunity for a more individualized treatment approach.
